# Primary Gastric Malignant Melanoma

**DOI:** 10.7759/cureus.11792

**Published:** 2020-11-30

**Authors:** Konstantinos Kosmas, Ioannis Vamvakaris, Eleni Psychogiou, Eirini Klapsinou, Dimitra Riga

**Affiliations:** 1 Cytology Department, General Hospital of Thoracic Diseases of Athens “Sotiria”, Athens, GRC; 2 Pathology Department, General Hospital of Thoracic Diseases of Athens “Sotiria”, Athens, GRC; 3 Department of Cytology, Diagnostic and Therapeutic Center of Athens “Hygeia”, Athens, GRC

**Keywords:** primary gastric malignant melanoma, pgmm, immunohistochemistry, case report

## Abstract

Primary gastric malignant melanoma (PGMM) is an extremely rare clinical entity, and few cases have been described in the literature. Here, we report a histologically confirmed PGMM case of a 74-year-old man with a mass in the stomach found by gastroscopy. The patient had no history of melanoma. This rare disease may be misdiagnosed for another gastric malignant tumor type when there is no known primary lesion. Early detection and surgical intervention are critical for long term survival or cure, though the tumor is often advanced at the time of diagnosis and is associated with a dismal outcome.

## Introduction

Worldwide, malignant melanoma is one of the most aggressive and treatment-resistant skin cancers, with 287,700 new cases and 60,700 deaths in 2018 [1]. While melanoma is predominantly a cutaneous cancer, extracutaneous melanoma tumor does occur and encompasses ocular, mucosal, and leptomeningeal melanomas with an incidence of fewer than 1/100,000 person-years [2]. Mucosal melanomas comprise approximately 1.3% of all melanocytic malignancies [3]. Primary gastric malignant melanoma (PGMM), a subtype of mucosal melanoma, is extremely rare [4]. So far, in the literature, 25 cases have been reported worldwide [5]. PGMM has been reported to occur in older patients with an average age of 71 years, often at an advanced stage, and is characterized by a worse prognosis [2] due to the tendency to grow faster and more aggressively than metastatic tumors, perhaps due to the rich lymphovascular supply available in the intestinal mucosa [6]. PGMM clinical manifestations are similar to other upper gastrointestinal (GI) lesions, such as weight loss, abdominal pain, anemia, nausea, and melaena due to GI bleeding [4,7]. Histologic diagnosis of melanoma requires the exclusion of metastasis from a primary cutaneous or ocular lesion, with full skin and eye examination by a dermatologist and an ophthalmologist, to rule out another primary source, as this influences both the prognosis and further management of the patient, including whether surgical resection is advisable [4]. The extremely low incidence and lack of awareness of this disease may also contribute to the misdiagnosis by phy­sicians and pathologists, especially in cases lacking a known primary lesion and when the tumor tissue excised with biopsy contains no melanin pig­mentation, such as the one we present. Early detection and surgical intervention are critical for long-term survival, though the overall prognosis is very poor.

A part of this case report was presented as a brief abstract in the 36th Balkan Medical Week on September 25-26, 2020 in Bucharest, Romania (Abstract: Riga D, Vamvakaris I, Kosmas K, Michelis V, Konstantinidou A, Psychogiou E. IM9. Primary Gastric Malignant Melanoma (PGMM). The 36th Balkan Medical Week; September 25-26, 2020).

## Case presentation

**Figure 1:**
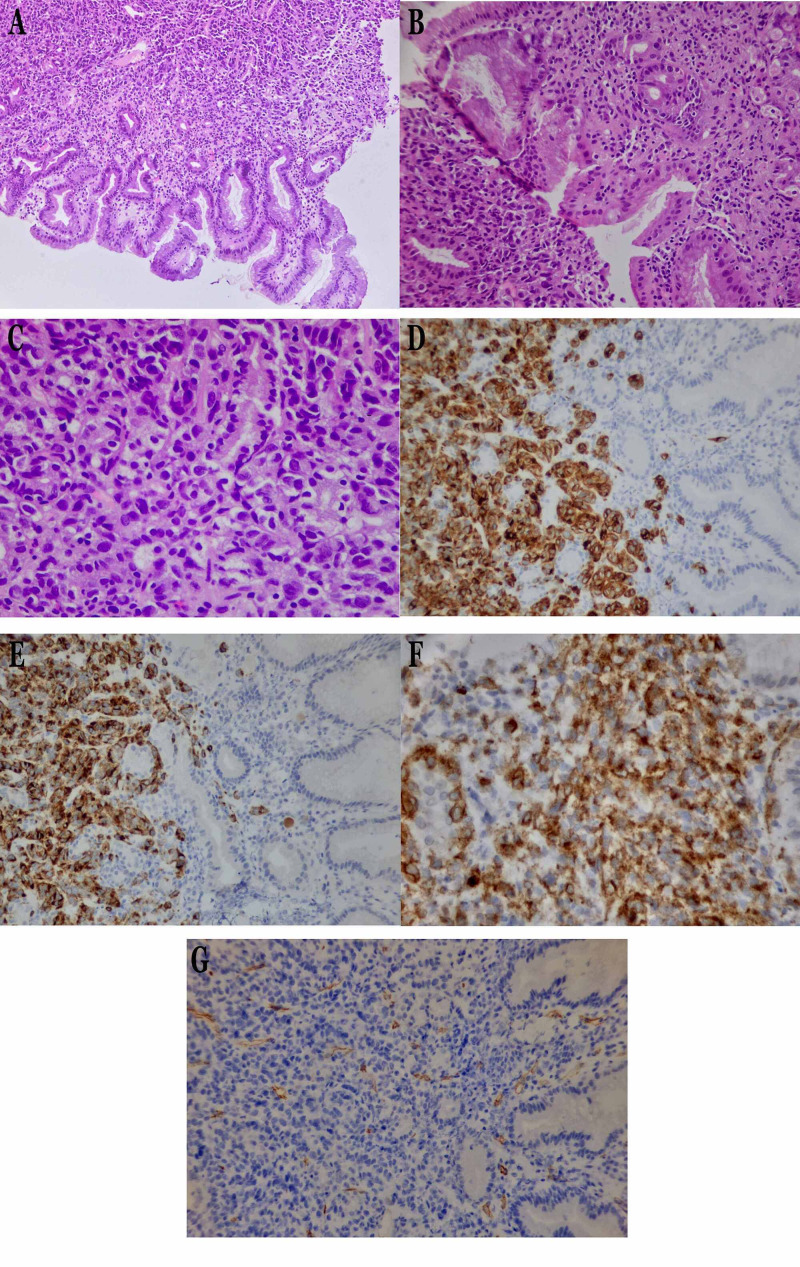
Hematoxylin and eosin staining and immunohistochemical expression by the tumor cells (A) Infiltration of the gastric mucosa by a tumor consisting of atypical epithelioid or spindle cells (hematoxylin and eosin staining, x20). (B) The tumor cells were round with hyperchromatic eccentric nuclei and high nuclear/cytoplasmic ratio, rare nucleoli, and mixed eosinophilic and basophilic cytoplasm (hematoxylin and eosin staining, x40). (C) The tumor cells appeared dyscohesive with no identifiable pigment formation (hematoxylin and eosin staining, x200). Immunohistochemical expression of (D) Melan-A(+) x200, (E) HMB-45(+) x200, (F) CD117(+) x400, and (G) CD34(-) x200 by the tumor cells.

A 74-year-old male patient was admitted to our hospital with a history of fatigue, loss of appetite, and dark tarry stools for more than 15 days. Medical history included arterial hypertension and smoking. High-resolution computed tomography (HRCT) revealed an ulcerated mass in the gastric cardia measuring 4.1 x 3.8 cm. Gastroscopy was performed, and four grayish-white tissue samples, measuring 0.2 to 0.4 cm were sent for histologic examination. Microscopic examination revealed a tumor consisting of large atypical epithelioid or spindle cells growing in solid sheets and fascicles. The tumor cells were round, with hyperchromatic eccentric nuclei, high nuclear/cytoplasmic ratio, rare nucleoli, and mixed eosinophilic and basophilic cytoplasm. They appeared dyscohesive with no identifiable pigment formation. There was widespread mitotic activity and necrosis. Immunohistochemically the tumor cells were CK7(-), CD34(-) LCA(-) and CD117(+), HMB-45(+) and Melan-A(+) (Figure 1). Thorough clinical examination for possible sites of primary malignant melanoma (skin, mucous membranes, scalp, palms, eyes, genitals) was negative. Based on these results, a diagnosis of PGMM was established. The patient underwent six months of chemotherapy/immunotherapy and is still on strict follow-up.

## Discussion

Melanoma is an aggressive type of cancer that commonly presents in tissues where melanocytes reside. Most melanomas are cutaneous, but non-cutaneous tumors such as ocular, oral, nasopharyngeal, esophageal, bronchial, leptomeningeal, vaginal, anorectal, and nail-bed melanomas occur, although very rarely [6]. In addition to these sites, melanocytes have been shown to exist in the stomach, small intestine, and colon, which normally are devoid of melanocytes [8]. A systematic review done by Schizas et al. reviewed 25 PGMM cases. The neoplasms were located at the body of the stomach (54.2%), cardia (20.8%), antrum (16.7%), and fundus (8.3%) in descending order of frequency (Table 1) [5].

**Table 1:** Location of PGMMs *Tumor location data were not available for one case. The mean age was 63.4 ± 8.97 years (mean ± standard deviation).

Location	Total, n (%)
Body	13 (54.2)
Cardia	5 (20.8)
Antrum	4 (16.7)
Fundus	2 (8.3)
Total	24* (100)

The development and pathogenesis of primary melanoma within the GI tract are unknown, and two mechanisms have been proposed. First, the differentiation of amine precursor uptake and decarboxylation (APUD) cells to melanocytes since derivatives of the neural crest can maintain the capacity for differentiation and consequently undergo malignant transformation. Second, ectopic migration of melanocytic precursors suggested by the observation of benign melanosis in neoplasms of the GI tract [9]. Despite these theories, the cause of primary GI melanomas remains a mystery. Additionally, the GI tract is a common site of cutaneous melanoma metastasis. For this reason, diagnostic criteria for the distinction between primary GI mucosal melanoma and melanoma metastatic to the GI tract have been proposed to preclude this possibility. The diagnostic criteria for primary gastric melanoma include a histologically proven single lesion of melanoma in the stomach, no concurrent lesions anywhere else in the body, no history of melanoma, and disease-free survival of at least 12 months after curative surgery [10]. Histopathologic diagnosis of melanoma depen­ds on the identification of melanin in the cyto­plasm. Microscopically, most of the typical advanced melanomas are built up in a complex pattern, in which the tumor is composed of dif­ferent cell types, such as spindle, plasmacy­toid, and epithelioid tumor cells. They are arranged in a sheet-like, organoid/alveolar, neurotropic, or desmoplastic configuration [8]. Immunohistochemistry with some specific markers such as S-100, tyrosinase, Melan-A (MART-1), HMB-45 and SOX-10 [5,8,9] is usually required to confirm the diagnosis since a portion of mela­noma contains little or no melanin pigment and may mislead the pathologist to the diagnosis of other tumors in GI tract, such as lymphoma, poorly differenti­ated carcinoma, neuroendocrine tumors (NETs) and gastrointestinal stromal tumors (GISTs) [8]. The clinical manifestations of primary gastric mela­noma are similar to those of other gastric tumors, with weight loss, upper gastrointestinal bleeding, and anemia as the most common symptoms. Most patients are asymp­tomatic until the tumor becomes advanced. Computed tomography scan of the abdomen, upper endoscopy, and subsequent biopsy are the main diagnostic modalities [11]. There is no standard protocol for treatment due to few cases reported in the literature. Tumor resection has the best results in reducing symptoms and improving survival. The role of adjuvant chemothera­py and radiotherapy has not been fully elucidat­ed on this disease, though most of the cases in the literature suggested systemic chemotherapy following surgery [8]. The prognosis of primary gastric melanoma is extremely poor even compared to other primary melanomas of the GI tract, with a median survival time of five months, whereas the overall median survival for all primary gastrointestinal tract melanomas is 17 months [12]. The 5-year survival rate is only 3% [2].

## Conclusions

PGMM is an extremely rare disease with aggressive behavior and poor overall prognosis, mostly due to late diagnosis. The delay in the diagnosis of these cases is caused by non-specific clinical manifestations and easier metastasis due to the rich lymphatic and vascular supply of the gastrointestinal mucosa. Differential diagnosis between primary and metastatic lesions is essential. PGMM is a challenging diagnostic entity that requires thorough histopathological and clinical diagnostic investigation as it can be underdiagnosed, especially when there is no primary lesion and when the tumor tissue excised with biopsy contains no melanin pig­mentation, such as the one in our case, unless immunohistochemical stains for S-100 protein, Melan-A, HMB-45, and SOX-10 antibodies are positive. Early detection and surgical intervention are considered critical for improving survival, even if there is no clear consensus on the management of PGMM.
